# 1,2-Bis(1*H*-pyrrol-2-ylmethyl­ene)diazane monohydrate

**DOI:** 10.1107/S1600536809025422

**Published:** 2009-07-08

**Authors:** Lin Yan, Hong Zhao, Chun-Ling Chen

**Affiliations:** a Institute of Pharmacy, Henan University, Kaifeng 475004, People’s Republic of China; b Key Laboratory of Natural Medicine and Immunal Engineering, Henan University, Kaifeng 475004, People’s Republic of China; c Henan Chemical Industry Senior Technician School, Kaifeng 475001, People’s Republic of China; d Institute of Molecular and Crystal Engineering, College of Chemistry and Chemical Engineering, Henan University, Kaifeng 475004, People’s Republic of China

## Abstract

The mol­ecular structure of title compound, C_10_H_10_N_4_·H_2_O, has an inversion centre located on the mid-point of the N—N bond of the mol­ecule. A twofold rotation axis passes through the water O atom. In the crystal structure, a two-dimensional network is constructed through N—H⋯O and O—H⋯N hydrogen bonds.

## Related literature

For the biological properties of azines, see: Khodair & Bertrand (1998 ▶). For their potential applications, see: Espinet *et al.* (1998 ▶); Nalwa *et al.* (1993 ▶); Schweizer *et al.* (1993 ▶).
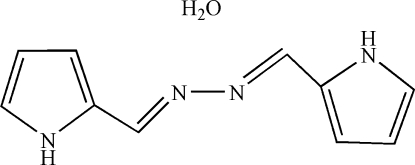


## Experimental

### 

#### Crystal data

C_10_H_10_N_4_·H_2_O
*M* *_r_* = 204.24
Monoclinic, 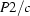

*a* = 12.006 (4) Å
*b* = 6.5806 (19) Å
*c* = 6.914 (2) Å
β = 105.253 (6)°
*V* = 527.0 (3) Å^3^
*Z* = 2
Mo *K*α radiation
μ = 0.09 mm^−1^
*T* = 296 K
0.23 × 0.17 × 0.10 mm

#### Data collection

Bruker SMART CCD area-detector diffractometer
Absorption correction: multi-scan (*SADABS*; Sheldrick, 2001 ▶) *T*_min_ = 0.980, *T*_max_ = 0.991
2143 measured reflections
910 independent reflections
583 reflections with *I* > 2σ(*I*)
*R*_int_ = 0.052

#### Refinement

*R*[*F* ^2^ > 2σ(*F* ^2^)] = 0.065
*wR*(*F* ^2^) = 0.191
*S* = 1.05
910 reflections
74 parameters
1 restraint
H atoms treated by a mixture of independent and constrained refinement
Δρ_max_ = 0.24 e Å^−3^
Δρ_min_ = −0.16 e Å^−3^

### 

Data collection: *SMART* (Bruker, 2001 ▶); cell refinement: *SAINT-Plus* (Bruker, 2001 ▶); data reduction: *SAINT-Plus*; program(s) used to solve structure: *SHELXS97* (Sheldrick, 2008 ▶); program(s) used to refine structure: *SHELXL97* (Sheldrick, 2008 ▶); molecular graphics: *PLATON* (Spek, 2009 ▶); software used to prepare material for publication: *PLATON*.

## Supplementary Material

Crystal structure: contains datablocks global, I. DOI: 10.1107/S1600536809025422/at2834sup1.cif

Structure factors: contains datablocks I. DOI: 10.1107/S1600536809025422/at2834Isup2.hkl

Additional supplementary materials:  crystallographic information; 3D view; checkCIF report

## Figures and Tables

**Table 1:** Hydrogen-bond geometry (Å, °)

*D*—H⋯*A*	*D*—H	H⋯*A*	*D*⋯*A*	*D*—H⋯*A*
N1—H1*A*⋯O1*W*	0.86	2.07	2.910 (3)	167
O1*W*—H1*W*⋯N2^i^	0.826 (10)	2.132 (16)	2.917 (3)	159 (4)

Symmetry code: (i) 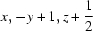.

**Fig. 1.**
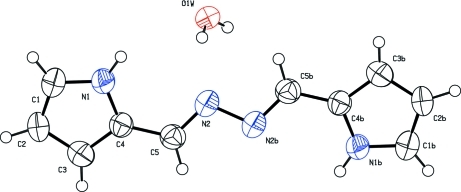
The molecular structure of (I), showing the atom-labelling scheme. Displacement ellipsoids are drawn at the 50% probability level.

**Fig. 2.**
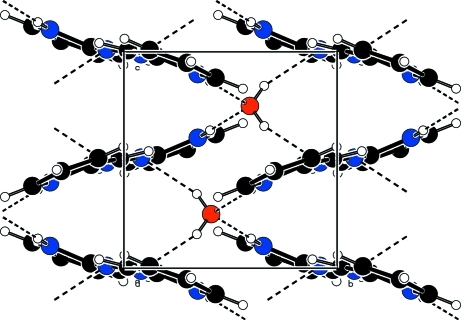
Two-dimensional structure of (I) along [100] direction. Hydrogen bonds are shown in the dashing line.

## supplementary crystallographic information

### Comment

Recently, dinucleating diazine ligands containing a single N—N bond have received considerable attention due to their biological properties (Khodair, *et al.* 1998), their potential applicability in bond formations (Schweizer, *et al.*, 1993), the design of liquid crystals (Espinet, *et al.*, 1998) as well as non-linear optical materials (Nalwa, *et al.*, 1993). we now report the structure of the title compound, (I).

Compound (I) consists of a 1,2-bis((1*H*-pyrrol-2-yl)methylene)hydrazine organic molecule and a crystal water molecule (Fig.1). The molecular structure of title compound has an inversion centre located on the midpoint of the N—N bond of the molecule. A two-fold rotation axis pass through the water O atom. The N1/C1–C4 ring in (I) is coplanar, in which the C–N bond distances range from 1.344 (4) to 1.377 (4) Å. However, C5—N2 [1.308 (4) Å] is typical for a C═N double bond. The N2—N2b bond distance is 1.395 (5), indicating a N—N single bond.

Two intra and intermolecular hydrogen bonds N—H···O and O—H···N (Table 1) help to establish the molecular conformation, and constructing infinite two-dimensional network along [100] plane (Fig. 2).

### Experimental

An ethanol solution containing hydrazine hydrate (0.20 g, 4 mmol) was added dropwise with constant stirring and slow heating to a solution of pyrrole-2-carboxaldehyde (0.38 g, 4 mmol) in the same solvent with five drops of acetic acid. The solution was refluxed for 2 h. Then the resultant solution was filtered. Red crystals suitable for X-ray studies were obtained by slow evaporation of the ethanol solution [yield: 65%].

### Refinement

The water H atom was found from a difference Fourier map and refined freely. Other H atoms were treated as riding, with C—H distances of 0.93 Å and N—H distances of 0.86 Å, and were refined as riding with *U*_iso_(*H*)=1.2*U*_eq_(C and N).

### Crystal data

**Table:**

C_10_H_10_N_4_·H_2_O	*F*(000) = 216
*M**_r_* = 204.24	*D*_x_ = 1.287 Mg m^−^^3^
Monoclinic, *P*2/*c*	Mo *K*α radiation, λ = 0.71073 Å
Hall symbol: -P 2yc	Cell parameters from 519 reflections
*a* = 12.006 (4) Å	θ = 3.1–23.4°
*b* = 6.5806 (19) Å	µ = 0.09 mm^−^^1^
*c* = 6.914 (2) Å	*T* = 296 K
β = 105.253 (6)°	Block, red
*V* = 527.0 (3) Å^3^	0.23 × 0.17 × 0.10 mm
*Z* = 2	

### Data collection

**Table:**

Bruker SMART CCD area-detector diffractometer	910 independent reflections
Radiation source: fine-focus sealed tube	583 reflections with *I* > 2σ(*I*)
graphite	*R*_int_ = 0.052
φ and ω scans	θ_max_ = 25.1°, θ_min_ = 1.8°
Absorption correction: multi-scan (*SADABS*; Sheldrick, 2001)	*h* = −14→12
*T*_min_ = 0.980, *T*_max_ = 0.991	*k* = −7→7
2143 measured reflections	*l* = −8→6

### Refinement

**Table:**

Refinement on *F*^2^	Secondary atom site location: difference Fourier map
Least-squares matrix: full	Hydrogen site location: inferred from neighbouring sites
*R*[*F*^2^ > 2σ(*F*^2^)] = 0.065	H atoms treated by a mixture of independent and constrained refinement
*wR*(*F*^2^) = 0.191	*w* = 1/[σ^2^(*F*_o_^2^) + (0.1036*P*)^2^] where *P* = (*F*_o_^2^ + 2*F*_c_^2^)/3
*S* = 1.05	(Δ/σ)_max_ < 0.001
910 reflections	Δρ_max_ = 0.24 e Å^−^^3^
74 parameters	Δρ_min_ = −0.15 e Å^−^^3^
1 restraint	Extinction correction: *SHELXL97* (Sheldrick, 2008), Fc^*^=kFc[1+0.001xFc^2^λ^3^/sin(2θ)]^-1/4^
Primary atom site location: structure-invariant direct methods	Extinction coefficient: 0.06 (2)

### Special details

**Table:**

Geometry. All e.s.d.'s (except the e.s.d. in the dihedral angle between two l.s. planes) are estimated using the full covariance matrix. The cell e.s.d.'s are taken into account individually in the estimation of e.s.d.'s in distances, angles and torsion angles; correlations between e.s.d.'s in cell parameters are only used when they are defined by crystal symmetry. An approximate (isotropic) treatment of cell e.s.d.'s is used for estimating e.s.d.'s involving l.s. planes.
Refinement. Refinement of *F*^2^ against ALL reflections. The weighted *R*-factor *wR* and goodness of fit *S* are based on *F*^2^, conventional *R*-factors *R* are based on *F*, with *F* set to zero for negative *F*^2^. The threshold expression of *F*^2^ > σ(*F*^2^) is used only for calculating *R*-factors(gt) *etc*. and is not relevant to the choice of reflections for refinement. *R*-factors based on *F*^2^ are statistically about twice as large as those based on *F*, and *R*- factors based on ALL data will be even larger.

### Fractional atomic coordinates and isotropic or equivalent isotropic displacement parameters (Å^2^)

**Table:**

	*x*	*y*	*z*	*U*_iso_*/*U*_eq_	
C1	0.1938 (3)	0.5705 (6)	0.1197 (5)	0.0584 (11)	
H1B	0.1862	0.4418	0.1702	0.070*	
C2	0.1062 (3)	0.7050 (6)	0.0509 (6)	0.0622 (11)	
H2C	0.0287	0.6846	0.0448	0.075*	
C3	0.1546 (3)	0.8794 (5)	−0.0091 (6)	0.0614 (11)	
H3A	0.1152	0.9973	−0.0607	0.074*	
C4	0.2711 (3)	0.8453 (5)	0.0221 (5)	0.0456 (9)	
C5	0.3587 (3)	0.9809 (5)	−0.0076 (5)	0.0489 (9)	
H5A	0.3398	1.1158	−0.0408	0.059*	
N1	0.2931 (2)	0.6536 (4)	0.1029 (4)	0.0499 (9)	
H1A	0.3597	0.5962	0.1372	0.060*	
N2	0.4646 (2)	0.9192 (4)	0.0110 (4)	0.0481 (8)	
O1W	0.5000	0.4090 (5)	0.2500	0.0531 (10)	
H1W	0.487 (3)	0.342 (5)	0.343 (4)	0.055 (11)*	

### Atomic displacement parameters (Å^2^)

**Table:**

	*U*^11^	*U*^22^	*U*^33^	*U*^12^	*U*^13^	*U*^23^
C1	0.056 (2)	0.056 (2)	0.065 (2)	−0.0124 (18)	0.0201 (17)	0.0022 (18)
C2	0.041 (2)	0.064 (2)	0.084 (3)	−0.0099 (17)	0.0200 (19)	0.000 (2)
C3	0.050 (2)	0.056 (2)	0.079 (3)	0.0044 (17)	0.0179 (18)	−0.0031 (19)
C4	0.0428 (18)	0.0454 (18)	0.0471 (19)	−0.0032 (15)	0.0095 (14)	−0.0033 (15)
C5	0.048 (2)	0.0484 (19)	0.050 (2)	0.0017 (15)	0.0106 (15)	0.0009 (16)
N1	0.0400 (16)	0.0477 (17)	0.0600 (19)	−0.0018 (12)	0.0099 (13)	0.0041 (14)
N2	0.0531 (18)	0.0462 (16)	0.0456 (17)	−0.0090 (12)	0.0142 (13)	−0.0017 (13)
O1W	0.051 (2)	0.0377 (19)	0.074 (3)	0.000	0.0219 (19)	0.000

### Geometric parameters (Å, °)

**Table:**

C1—N1	1.344 (4)	C4—N1	1.377 (4)
C1—C2	1.361 (5)	C4—C5	1.435 (5)
C1—H1B	0.9300	C5—N2	1.308 (4)
C2—C3	1.397 (5)	C5—H5A	0.9300
C2—H2C	0.9300	N1—H1A	0.8600
C3—C4	1.376 (5)	N2—N2^i^	1.395 (5)
C3—H3A	0.9300	O1W—H1W	0.826 (10)
			
N1—C1—C2	109.1 (3)	C3—C4—C5	129.0 (3)
N1—C1—H1B	125.5	N1—C4—C5	123.9 (3)
C2—C1—H1B	125.5	N2—C5—C4	121.5 (3)
C1—C2—C3	107.1 (3)	N2—C5—H5A	119.2
C1—C2—H2C	126.4	C4—C5—H5A	119.2
C3—C2—H2C	126.4	C1—N1—C4	109.1 (3)
C4—C3—C2	107.7 (3)	C1—N1—H1A	125.4
C4—C3—H3A	126.1	C4—N1—H1A	125.4
C2—C3—H3A	126.1	C5—N2—N2^i^	110.9 (3)
C3—C4—N1	106.9 (3)		

Symmetry codes: (i) −*x*+1, −*y*+2, −*z*.

### Hydrogen-bond geometry (Å, °)

**Table:**

*D*—H···*A*	*D*—H	H···*A*	*D*···*A*	*D*—H···*A*
N1—H1A···O1W	0.86	2.07	2.910 (3)	167
O1W—H1W···N2^ii^	0.83 (1)	2.13 (2)	2.917 (3)	159 (4)

Symmetry codes: (ii) *x*, −*y*+1, *z*+1/2.
